# The Role of Anthropometry in Decision-Making for Injury Prevention Among Elite Flag Football Players

**DOI:** 10.3390/sports14040140

**Published:** 2026-04-01

**Authors:** Luis Gerardo Vázquez-Villarreal, Luis Felipe Talavera-Hernández, Martha Patricia Dergal-Irigoyen, Claudia Maceroni, Eleanor Louise Travis-Carr, José Miguel Martínez-Sanz, Nidia Rodriguez-Sanchez

**Affiliations:** 1 Medical Direction and Applied Sports Sciences, LFA Professional American Football League, Mexico City 01219, Mexico; gerardo.vazquez@lfa.mx; 2 Department of Nutrition, Faculty of Nursing and Nutrition, Autonomous University of Chihuahua, Chihuahua 31125, Mexico; 3 ABC Medical Center, Mexico City 05300, Mexico; marthadergal@gmail.com; 4 Sports Science Research, Seattle, WA 98103, USA; claudia@threebalance.com; 5 Musculoskeletal Health Research Group, School of Health, Leeds Beckett University, Leeds LS1 3HE, UK; e.travis@leedsbeckett.ac.uk; 6 Research Group in Applied Dietetics, Nutrition and Body Composition (DANuC), Department of Nursing, Faculty of Health Sciences, University of Alicante, 03690 Alicante, Spain; josemiguel.ms@ua.es; 7 Faculty of Health Sciences and Sport, University of Stirling, Scotland FK9 4LA, UK; nidia.rodriguezsanchez@stir.ac.uk

**Keywords:** anthropometric characteristics, body composition, injury prevention, flag football, ACL injury, hand injury

## Abstract

Although Flag Football (FF) is growing worldwide, the literature to guide sports sciences in preventing injuries is scarce. The aim of this study was to analyse how anthropometric characteristics were associated with injury in elite FF players. Athletes completed a full profile according to the International Society of Advances in Kinanthropometry (ISAK), including weight, height, sitting height, arm span length, skinfolds, girths, length and breadth bones, and an injury questionnaire was administered. Logistic regression models and Receiver Operating Characteristic (ROC) curves were conducted. In total, 108 FF national team players, 34 female (26.7 ± 4.3 years old) and 74 male (26.9 ± 5.1 years old), participated. Of these, 62% FF players reported injuries. Relaxed arm and flexed and contracted arm girths are related to increased or reduced injury risks (Odds = 2.932, *p* = 0.008; Odds = 0.335, *p* = 0.009, respectively), while longer tibia length and higher muscle mass also increase the risk (Odds = 1.407, *p* = 0.034; Odds = 1.223, *p* = 0.010, respectively). Specific cut-off points were defined by sex, such as hip circumference, established at 103 cm in the Anterior Cruciate Ligament (ACL) model for males, increasing the risk by 5 times. Anthropometric characteristics were related to injury incidence and could be used by sports science practitioners as an efficient decision-making tool to describe and analyse the static and dynamic components of FF players in injury prevention.

## 1. Introduction

The game of Flag Football (FF) is a variant of American tackle football (TF) characterised by the absence of direct physical contact [1,2]. In recent years, it has gained such popularity (both recreationally and competitively) that the International Olympic Committee has approved its inclusion at the Los Angeles Olympic Games in 2028 [3,4].

FF maintains some rules from TF, allowing the practice of strategic thinking and other skills such as throwing, catching, running and making quick changes in direction [5]. Despite its non-contact nature and rules which are designed to reduce contact [2], FF is not exempt from physical risks. To reduce the physical nature of blocking and tackling in TF, FF players wear flags attached to their waists that the opponents pull to make a tackle [2]. In contrast to TF, a smaller playfield in FF might promote higher-velocity gameplay; as a result, FF players are exposed to various physical demands that involve various explosive movements and changes of direction with large dynamic loads on the joints, acceleration, deceleration, change of direction tasks and jumping mechanisms, increasing their risk of injuries due to the static and dynamic instability of the knee joint [6,7,8].

The role of medical teams in preventing injuries has evolved dramatically in the last decade. By increasing the awareness of injuries in elite sports, the core focus of medical teams has shifted from a reactive response to a preventive approach [5]. This transition of focus requires the development of strategies based on data collection, analysis of injury trends, and a systematic approach that involves an interdisciplinary collaboration between coaches and medical practitioners, which would include athletic trainers, doctors, physiotherapists, nutritionists, and sports scientists [9]. This change of paradigm in the role of sport science and medical support teams enhances the longevity of athletes, especially when they are exposed to preventive strategies in the early years of sports practice [10,11], justifying the importance of integrating sport science and medical support teams as fundamental in high-performance sports.

In high-performance sports, understanding the factors that contribute to injury is essential for developing effective prevention strategies [12]. FF is typically observed as the safer version of TF, but studies have reported that injury rates per exposure may be higher in FF, although less severe [13]. In FF, the most frequently reported injury locations are hand, shoulder and knee [6]. ACL injuries in female sports could be particularly important to describe and explore since there are factors that potentially increase the risk of injuries such as the oestrogen load in the preovulatory stage [14]. Several studies have shown that anthropometric characteristics—such as body composition, body mass index (BMI), lean mass, trophism (the state of muscle development) and body fat distribution—can influence injury susceptibility, considering the stability of the Centre of Mass (CoM) as an injury factor [15]. It could be a common mistake to assume anthropometrics only as a body composition parameter, but there are other applications of bone indexes that could be associated with biomechanics combinations such as levers involved directly in torque forces.

Association models of sports injuries to develop preventive therapeutic applications for medicine, athletic training, and physical conditioning are fundamental. The goal is to analyse the likelihood of injury and also allow for personalised interventions with athletes to prevent injuries [16]. In other sports, such as TF, neuromuscular training programmes have shown effectiveness trough proprioception, balance and strength exercises, reducing the risk of non-contact knee and ACL injuries [17,18]. Although factors such as age, height, and BMI have been studied in youth FF players, results have not been conclusive, linking these anthropometric factors to injury incidence [19]. Therefore, there is a need to add anthropometric characteristics and indexes to strengthen and increase the specificity and sensitivity of these predictive and preventive models.

To identify injury patterns in FF, the current study was conducted during the 2023 International Federation of American Football (IFAF) European Flag Football Championship. It aimed to analyse the association between the anthropometric characteristics of elite athletes and the prevalence of injuries, while also considering the preventive actions adopted by the athletes. This research seeks to provide empirical evidence to guide future injury-prevention interventions in this discipline. The main hypothesis of this study is that specific anthropometric lengths and diameters are associated with injury incidence through biomechanically conditioned bone and body composition structures.

## 2. Materials and Methods

A cross-sectional observational study was conducted to identify how anthropometrics, strength, power and preventive medicine strategies are related to injury incidence. The study was conducted with FF national team players from European and Asian countries competing at the IFAF European Flag Football Championship 2023 at the University of Limerick, Ireland. The University of Stirling NHS, Invasive or Clinical Research (NICR) Committee granted ethical approval (Ref: NICR 2023 14791 10383). The research design followed the World Medical Association codes and the Helsinki Declaration [20]. In addition, the study design and development of the manuscript followed the STROBE statement [21].

A total sample of 139 FF players, 48 female (26.6 ± 4.5 years old) (e.g., mean ± SD) and 91 male (26.6 ± 5.3 years old), were evaluated. The participants represent nine European and Asian nations (Germany, Great Britain, France, Ireland, Spain, Israel, Georgia, Sweden, and Poland). The population was selected by non-probabilistic convenience sampling. The sample was recruited by inviting the coaches of all participating teams. They were informed about the study’s characteristics, the objectives and method of the research, and invited to participate. Once they agreed to participate, all the players signed the informed consent before starting the research.

Sample size calculation was performed with Rstudio software (version 3.15.0, Rstudio Inc., Boston, MA, USA). The significance level was set a priori at *p* = 0.05. The standard deviation (SD) was to the percentage of adipose tissue (AT) from previous studies (SD = 2.12) [22], with an estimated error (d) of 0.38%. Based on this methodology, the minimum required sample size was 120 participants, with an estimated error (d) of 0.5 for SD percentage within a 95% confidence interval (CI). The criteria for inclusion in the study were: (a) be healthy with medical authorization for the practice of federated sport; (b) training a minimum of 5 days per week. The exclusion criteria were: (a) being injured at the time of the evaluations; (b) having been denied by their team’s head coach to take part in the study. Finally, only 109 FF players, 34 female (26.7 ± 4.3 years old) and 74 male (26.9 ± 5.1 years old), completed the injuries questionnaire.

### 2.1. Anthropometric Measurements

Measurements were performed in accordance with the ISO 7250-1:2017 [23] and the International Society for the Advancement of Kinanthropometry (ISAK) full profile protocol standards for marking and locating [24]. Measurements were conducted by anthropometrists of level 2 or 3 accredited by ISAK in duplicate or triplicate with an inter-evaluator TEM of 0.09% for basic measurements, 2.98% for skinfolds and 0.88% for girths. A SECA 862 scale (SECA, Hamburg, Germany) with 100 g accuracy was used to measure body mass; a Seca 217 measuring stadiometer (SECA, Hamburg, Germany) with 1 mm accuracy was used to measure stretch height and sitting height; a wingspan meter (Smartmet, Jalisco, Mexico) with 1 mm accuracy was used to measure arm span; a metallic tape (Lukfin, Mexico) with 1 mm accuracy to measure girths; a small and large sliding calliper (Roscraft, Canada) with 1 mm accuracy to measure bone breaths; a segmometer (Holway, WI, USA) with 1 mm accuracy to measure length; and a Harpenden skinfold calliper (HaB Direct, Southam, UK) of 0.2 mm precision to measure skinfolds; and finally a wood box with a dimension of 30 × 40 × 50 centimetres (Nutriequipo, Jalisco, Mexico) was used to help the performance of the evaluation.

Ross and Kerr’s five-component model (adipose mass, muscle mass, bone mass, residual mass and skin) was estimated to assess body composition [25,26], as well as Adipose-Muscular Index, Muscular-Bone Index [27] and the sum of six and eight skinfolds [28]. Somatotype was estimated by the Heath–Carter method [29].

Additionally, anthropometric performance indices were calculated (Brachial Index, Crural Index, Relative Arm Span, Acromio-Iliac Index, Cormic Index, Lower Limb Relative Index, Arm Span–Height Difference, Active Body Mass Index (IAKS), Free Fat Mass Index, Pignet Constitutional Index, Skeletal Index, Chest Index, Relative Length of Upper Limb, Relative Arm Length, Relative Forearm Length, Relative Hand Length, Relative Thigh Length, Relative Leg Length, Relative Foot Length) [27,28].

### 2.2. Injury Evaluation

Data were collected via an online survey platform (Google Forms), added as Supplementary Material. Participants were provided with a QR code to the survey. The survey was written in English, and it was answered on individuals’ personal smartphones. The survey collected data on nationality, sex, playing position, and injury patterns, including years of experience, frequency and duration of physical training, frequency of implementing preventive measures to avoid injuries, type of injuries in the last 5 years, and location of those injuries. This was the first survey of its kind, and therefore some questions were designed according to the criteria of the research team, including sport medicine specialists and applied science practitioners, using the previous literature to inform them. Eleven questions were included in the final survey design: ten were categorical, with yes/no options, progressive options, type, location, and injury severity; three complementary open-text questions were included to collect detailed qualitative information. The survey can be found as Supplementary Materials.

### 2.3. Statistical Analysis

Descriptive data is presented as means and standard deviations for continuous variables. Categorical variables were analysed as frequencies and percentages. A *t*-test for means comparison was used to identify differences by sex. Multivariate analyses were conducted to evaluate the association between anthropometrics, strength, power, and preventive medicine actions and injury incidence using logistic regression models.

Single logistic regressions were first performed to examine potential associations. Multiple logistic regression analyses were conducted after plausibility criteria were met to examine relationships, adjusting for different variables or potential confounders. A forward stepwise regression model was applied to select the best models. Multicollinearity among independent variables was assessed using the variance inflation factor (VIF). Odds were reported according to logistic regression analysis.

ROC curves of each model were analysed, and variables were separated to evaluate the discriminative capacity of the models through the Area Under the Curve (AUC), which is classified as follows: 0–0.5, model without discrimination; 0.6–0.7, poor discrimination; 0.7–0.8, acceptable discrimination; 0.8–0.9, very good discrimination; and more than 0.9, excellent discrimination. Results from the ROC curves were also used to identify cut-off points using the complementary analysis of the Youden index, selecting the level of the variable with the highest sensitivity and specificity to predict injuries.

Significance was set to *p* ≤ 0.05 and a confidence interval (CI) of 95%. All descriptive calculations and means comparisons were performed using the Statistical Package for the Social Sciences v.18 (SPSS Inc.), and multiple regression analyses were developed using the STATA statistical package v.11 (Stata Corp., College Station, TX, USA).

## 3. Results

Anthropometric characteristics (skinfolds, perimeters, length and breadth bones), body composition (Ross and Kerr’s five-component model, as well as Adipose-Muscular Index, Muscular-Bone Index and the sum of six and eight skinfolds), somatotype and performance indexes (such as Brachial Index, Crural Index, Relative Arm Span, Acromio-Iliac Index, Cormic Index, etc.), show statistical differences between male and female players but not between playing position. Male players were reported to be heavier, taller, with less adiposity and more muscle mass than female players. All the specific results from the anthropometric characterisation are available in a previous publication but can also found in Supplementary Materials [30]. A total of 109 athletes (75 males and 34 females) met the criteria and answered the injury questionnaire to participate in the analysis of this study. Of the population, 62.6% reported having suffered an injury in the last 5 years (Table 1).

Males reported more injuries (68%) in proportion to females (52%); however, the injury location with more incidences was the hand for both sexes (see Table 1). Injury epidemiology was also analysed by player position (Table 2), with safety and rusher positions being the most injured. Shoulder injuries were the least reported overall, by sex and player position. Regarding ACL injuries, safety was the most injured position, reported by more than half of the players (54.5%), while receiver, centre, and defensive positions reported lower incidences (34%, 37.5%, and 35.7%, respectively). Hand injury incidences were reported at up to 80%, with rusher, safety, and defensive being the most injured positions (80%, 63.6%, and 52.4%, respectively).

To analyse the predictive capability of anthropometrics and other variables over injuries, a logistic multilinear model with forward steps was conducted (Table 3). Regarding playing positions, being a defensive back increases the risk of injuries 8 times (Odds = 8.203, *p* = 0.022) when compared to quarterbacks. Anthropometric characteristics such as relaxed arm and flexed and contracted arm girths show how local adiposity or muscle mass are related to increased or reduced injury risks in upper limbs (Odds = 2.932, *p* = 0.008; Odds = 0.335, *p* = 0.009, respectively). Regarding lower limbs, each increase in centimetres in tibia length also increases the risk of injury by 40% (Odds = 1.407, *p* = 0.034). To conclude, paradoxically, increases in muscle mass were associated with the risk of injury by 22% when the other variables are kept constant (Odds = 1.223, *p* = 0.010); as this do not seems plausible, this interpretation is not enough, probably because the variable of muscle mass expressed in a percentage has a critical point where an excess of muscle mass represents an increment of injury risk. Other anthropometric variables, performance indexes or body composition indexes were not shown to be mainly related to injuries; non-significant results are added as Supplementary Material.

**Figure 1:**
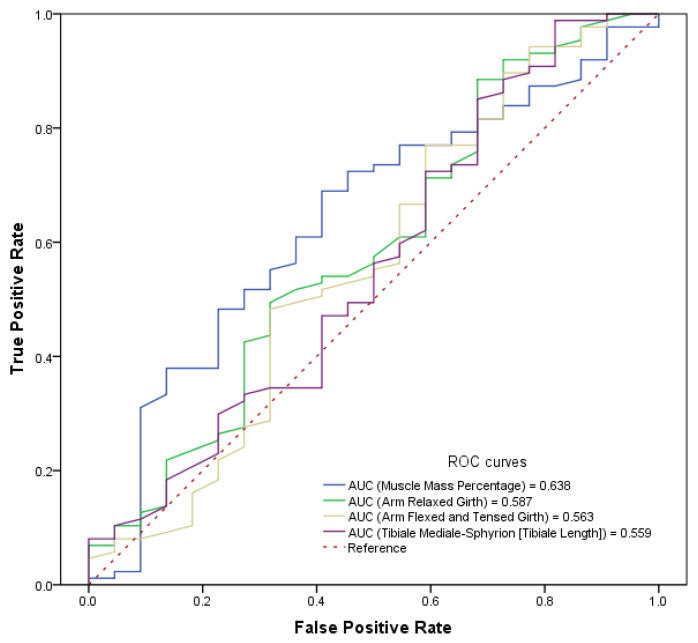
ROC curves and scores of anthropometric variables in relation to injuries.

The ROC curve of the model was analysed, and the area under the curve (AUC) was defined as 0.82, which means that the model has a very good discrimination capacity, when ROC curves were analysed for each variable individually. The Youden index indicated that 45.25% of muscle mass (*p* = 0.045) might be a cut-off point since the results are the best point of sensitivity and specificity to predict injuries (Figure 1). This means that over 45.25% of muscle mass is estimated by Kerr’s formula [25]; each increment represents a risk of injury in Flag Football (FF) players. It is important to note that the muscle bone index, adipose muscle index, sex, ankle girth, calf girth, radiale–stylion length, brachial index and bimalleolar breadth did not show a statistical correlation when the previous variables were added in the model.

However, due to sex differences found in the previous anthropometric characterisation [30], additional analyses by sex were performed. Male athletes’ injuries were related to some anthropometric characteristics previously described, such as relaxed and flexed arm girths and muscle mass percentage (Odds = 4.129, *p* = 0.009; Odds = 0.203, *p* = 0.007; Odds = 1.363, *p* = 0.007, respectively). The brachial index appears as a new predictive variable, indicating that each centimetre increase in forearm length over the arm length represents a 49% risk of injury (Odds = 1.493 *p* = 0.011). The model did not reveal any anthropometric association when analysed by female sex.

**Figure 2:**
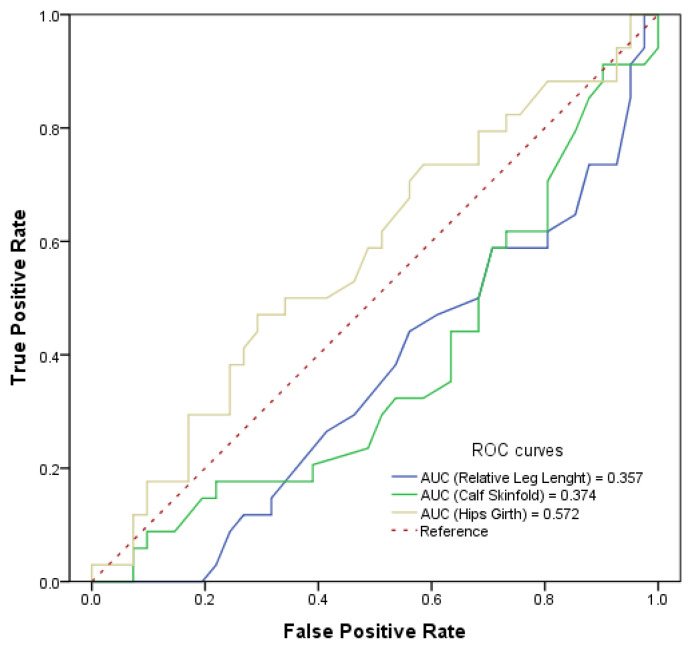
ROC curves and scores of anthropometric variables in relation to ACL injuries in males.

Specific models were developed using previous statistical strategies to explore anthropometric characteristics associated with ACL, shoulder, and hand injuries. Regarding ACL injuries, results in males (Table 4) show that a longer relative leg length reduces the risk of ACL injury by 58% (Odds = 0.418, *p* = 0.008). On the other hand, a greater hip girth increases the risk by more than 5 times when it is over 103 centimetres, in accordance with the ROC curves (Odds = 5.689, *p* = 0.010). In the same model, when the ROC curves were analysed (Figure 2), the calf skinfold cut-off point was defined at 7 mm (*p* = 0.044), indicating that a calf skinfold greater than 7 mm reduces the risk of injury by 86% (Odds = 0.137, *p* = 0.002). The complete model reported an AUC of 0.78.

Body mass, muscle mass, adipose muscular index and ankle girth did not show a statistical association when analysed in males. The ACL model in women indicates that each millimetre of calf skinfold reduces the risk of injury by 20% (Odd = 0.806, *p* = 0.024), while a wider hip girth represents an increased risk (Odds = 1.207, *p* = 0.027) with a cut-off point at 98 centimetres in accordance with the ROC curves. This model reports an AUC of 0.79, indicating acceptable discrimination.

Regarding shoulder injuries in males, a wider biacromial width represents a 35% decreased injury risk (Odds = 0.646, *p* = 0.011), while a longer iliospinale height increased the risk by 24% (Odds = 1.246, *p* = 0.004). On the other hand, only an increase in the subscapular skinfold in the female model was associated with a 24% increased risk (Odds = 1.242, *p* = 0.048). Both models had an AUC greater than 0.72.

**Figure 3:**
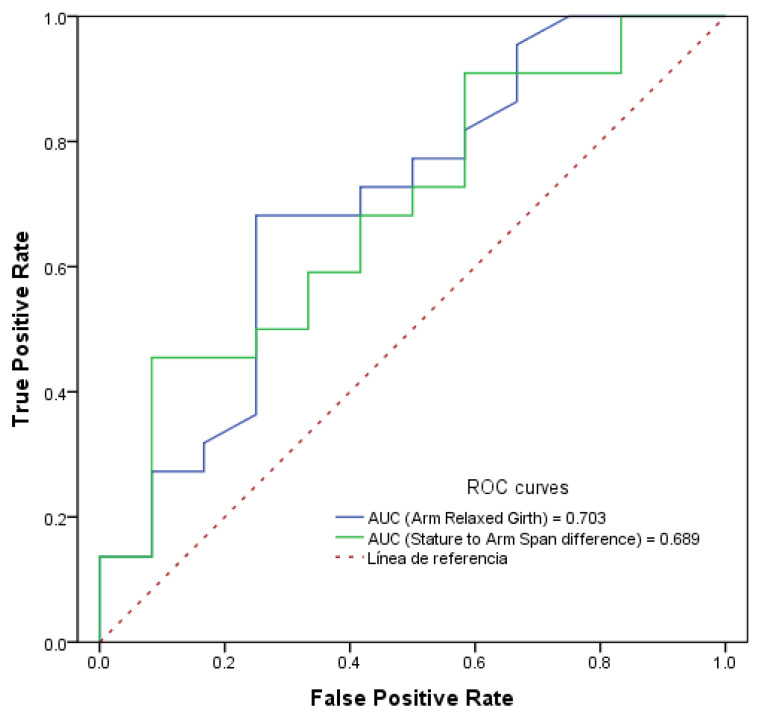
ROC curves and scores of anthropometric variables in relation to hand injuries in females.

Finally, when analysing hand injuries in females, as shown in Figure 3, the combination of a larger relaxed arm girth and a shorter arm span relative to stretch stature resulted in a higher risk of finger dislocation, fracture or sprain. After defining the cut-off points, the logistic model reported an association between a relaxed arm girth greater than 29.1 cm and an arm span relative to stature less than 1.5 cm, with a 7.6 and 12 times higher risk of injury respectively (Odds = 7.650, *p* = 0.027; Odds = 12.109, *p* = 0.041) when adjusted for the player’s position, reporting a model AUC of 0.85. In males, no association was observed between the anthropometric variables and hand injuries.

## 4. Discussion

This study analyses the epidemiology of injuries and how anthropometrics and other factors could be related to injury. The results report that more than 60% of the athletes were injured in the last 5 years before the data was collected, while most of them were located in the hand (47%), 34% were found to have sustained injury to the ACL, which is the most severe injury and most expensive to treat [31]. After analysis, the logistic regression model shows that applying preventive interventions in their sports medical follow-up were associated with reducing the probability of being injured by more than 80%, which means most of the injuries reported were preventable; a possible explanation is due to overload training or Relative Energy Deficiency (RED) for those non-contact injuries, as the International Olympic Committee suggest [32]. Anthropometrics were also associated with injuries, either as a protector or risk factors, depending on the biomechanical dynamic of the injury. Body composition and structural bones influence the stability of the Centre Of Mass (CoM) that could be modified (made up by static and dynamic components), creating a negative effect on the mechanical stability of different joints. Having a clear understanding of its influence could be paramount to prevent injuries [33,34,35,36].

Regarding anthropometrics as variables related to risk or preventive factors, we can have two different approaches: the structural components and the plastic, malleable components [37]. Those structural components cannot be modified as they are linked to genetics, such as bone lengths and breadths, stretch and sitting stature and arm span, which work as a solid structure and have conditioned the covering area increasing or decreasing the range; and those plastic components can be modified, such as skinfolds, girths and body mass, which are directly related to body composition [37]. More than accessibility to other body composition techniques such as bioimpedance or Dual Energy X-ray Absorptiometry (DEXA), anthropometric evaluation allows sports science practitioners to analyse how specific body segments could be involved in preventing injuries and increasing performance [29].

Regarding those solid anthropometric variables, in this study, some interesting characteristics were observed. With respect to ACL injuries, relative leg length in males was reported as a protective factor and could be associated with reducing risk by 58% for each additional centimetre. This could be explained by the dynamic forces related to deceleration mechanisms, since lengths not only mean a bigger brake leverage [38] but also performance and dynamic stability due to longer and more robust hamstrings and the soleus; transforming static anthropometric measures into dynamic, actionable insights for injury risk stratification thus are viable targets for preventive interventions [8,39].

By the same analogy, the risk of shoulder injuries is reported to be related to a reduction of 35% by each extra centimetre of bi-acromial breadth. On the shoulder, we observe an interesting secondary effect related to the CoM’s stability. As a higher iliospinale height is reported, a 24% increase in risk is associated. Additionally, while wider bone structure in the upper back is related to better mechanisms of shoulder control, reducing the risk of injury, when it is accompanied by longer lower limbs, an increase in torque or a twisting force tends to cause rotation and the probability of falling due to losing control [40].

One last structural finding in this study was associated with hand injuries. In females, a shorter arm span relative to stature, specifically, less than 1.5 cm, increases the probability of being injured by 12 times. This finding relates to the increased risk of colliding with other players, as reach on the ball is limited by a shorter arm span. This is different from the previous injuries because it is more related to a game dynamic established by a shorter arm span in female defensive players, limiting reach and the ability to capture the flag from opponents. Furthermore, this risk forces a defensive player to go inside the play with an increased risk of crashing into hands or fingers created by the rapid change of directions of offensive players’ hips, [2,7].

In contrast, plastic components of anthropometrics are related to the modification of muscle and adipose tissues, main components of body composition that depend on a complex balance of nutrition habits, workloads in training and genetics [37]. Anthropometric measurements and estimations allow us to analyse not only a global view but also specific segments of body composition. Susceptible areas can be detected for performing muscle trophism by increasing useful mass that hypothetically improves joint stability, as in studies where appendicular muscle mass estimated by DEXA was shown to be related to performance stability in young athletes [41].

When analysed by injury zone, this hypothetical relationship takes its own course, and in ACL injuries it works upside down. Paradoxically, extremely low adipose tissue results in an imbalance of knee joint stability. This study reports that a cut-off point of 7 mm of calf skinfold thickness was associated with an 86% reduction in injury risk in males, whereas a single millimetre increase in calf skinfold thickness in females was associated with a 20% protective factor. The literature on this is unclear, while higher adiposity is typically associated with a higher risk of lower limb injuries [42,43]. Most studies focus on BMI or abdominal obesity. However, a systematic review reveals that specific measures or adiposity distribution can also be related to a protective factor against injury, and a lower BMI could be related to higher bone stress [44]. A possible explanation of the results in this study focuses on the premise that the balance of useful mass depends not only on muscle trophism but also on adipose tissue, which plays a role in joint protection through the relationship of lower subcutaneous adipose tissue to REDs [45].

With regard to shoulder and hand injuries, an increase in subscapular skinfold and relaxed arm girth in females could be related to 24% and 7.6 times increased injury risks, respectively. There are specific trend zones of overstimulation (calf in both sexes) or infra stimulation (upper back and arms in females) by training loads that reflect specific body composition characteristics; in practical terms, it means that most of the time the practices are focused in the development of strength in lower limbs, as the skills of running are very important, but women do not prioritise working upper limbs as men usually do. These localized differences in muscle and adipose tissue were observed in this population [30]. These sex differences of risk factors associated with injuries also match those reported in other sports [46]. Once these systematic deficiencies in FF players are taken in account, training loads could be adjusted in order to minimise injuries, as is recommended by other authors who encourage new approaches in anthropometry applications in sports [47].

Additionally, it is important to understand the nature of the core fundamental movement in specific sports, since FF players use a lot of side cutting movements as natural and recurrent resources to drive the plays to end a down, as reported by Sankey et al. (2020); this kind of movements involves complex but precise movements that could reduce injury risk and perform a change of direction, such as foot placement on the ground, time of execution and hip acceleration to reduce destabilisation of the CoM [8]. This study also reports that wider hip girth was also associated with increases in the risk of injury by 5.6 times and 20% in males and females, respectively, establishing a cut-off point at 103 and 98 centimetres for males and females in this population. These findings add to other studies describing the anatomy of the hips in relation to sports performance [48]. It is important to note that these cut-off points reported were conditioned by anthropometric sex differences in this specific population, such as 17 kg of body mass and more than 15 cm of stretch stature between sexes [30].

## 5. Limitations

The first limitation of this study is the low and heterogeneous sample size, as discussed in the main publication [30]. The nature of the voluntary participation in the study results in a sample of players from different nations of Europe and Western Asian countries invited to the tournament, such as Israel and Georgia. However, this sample meets significance after statistical principles were applied. Although FF players assessed for this study represent the highest competitive level in Europe, they exclude the typical behavioural approach to injury prevention of other countries due to economic, sports infrastructural, and cultural differences. Despite these limitations, the approach of this study to link how both bone dimensions and body composition could be associated with injury prevention is the first reported at this competitive level.

Another limitation derived from the heterogeneous sample is the effect of the asymmetry of bone lengths over biomechanics loads as a potential risk factor in injury incidence. In this study, other side anthropometric measurements were unable to be performed. Although their own pathway to become part of the national team reduces the players’ body composition asymmetry through the years of experience and high training loads, the authors encourage further research to take into account the performance of both sides measurements to analyse symmetry.

The study design used for this research was cross-sectional by the nature of the event, although injury incidences were collected for the last years by a survey; this is a limitation on the research capability to establish a causal relationship, and potential recall bias must be considered. Logistical regression models and ROC curves were analysed to contribute to the proposal of specific values to help in the injury prevention protocols; however, longitudinal studies are recommended to add strong evidence to this proposal, specifically for the validation of the proposed cut-off points.

As this is an innovative approach to the use of anthropometric variables in decision-making protocols to prevent injuries, there is scarce evidence to compare results in this or other sports. The authors encourage researchers to replicate this statistical approach to increase the evidence-based protocols and clinical applications of anthropometrics in sports contexts.

## 6. Conclusions

The FF game’s nature has an injury risk relative to acceleration–deceleration in tight places, but this study explores a proposal as an alternative approach in decision-making for preventing injuries that must be validated. Considering sex differences in body composition and bone dimensions, a sex-specific analysis was needed. By analysing how anthropometrics could be associated with injury history, a proposal to guide sports medicine and sports science practitioners in the short, medium, and long term for injury prevention could be developed and tested.

Then, anthropometric measurements become an interesting approach to understanding movement dynamics and body composition of a specific location. Based on its analyses, sport- and player-specific positions and sport strategies can be developed, such as the cut-off points presented in this study that must be validated in a prospective study.

In order to transfer the knowledge presented in this study to different sports levels and sociocultural settings, although European athletes were considered in this paper, which are typically considered as from high-income countries, this knowledge, as a practical and accessible protocol, could be applied in different levels of sports development such as university teams or junior categories from different income settings with the purpose of strengthening their respective national teams. However, low- and middle-income countries must also be considered in further analyses to examine the impact of nutritional and sleep habits, accessibility to sports science practitioners, and infrastructure on injury epidemiology. By the nature of the elite FF players’ age, anthropometric measurements and injury evaluation do not take into account growth and development stages, which are a necessary next step, as other sports already rely on their programmes of repeated anthropometric measurements to evaluate and monitor rapid growth spurts that could compromise joint stability [10].

Finally, it is important to analyse how the anthropometric to injury relation reported in this study remains when the incidence of injury is tracked during a tournament, especially in females, when the menstrual stage is used to adjust the association due to the inflammatory process that occurs in different stages of the menstrual cycle [49].

## Figures and Tables

**Table 1:** Self-reported injuries in Flag Football players.

	Total (n = 109)	Male (n = 75)	Female (n = 34)
	n (%)	n (%)	n (%)
Injuries	87 (62.6)	62 (68.1)	25 (52.1)
ACL injuries	48 (34.5)	34 (37.4)	14 (29.2)
Shoulder injuries	34 (24.5)	25 (27.5)	9 (18.8)
Hand injuries	66 (47.5)	44 (48.2)	22 (45.8)

ACL = Anterior Cruciate Ligament.

**Table 2:** Injuries in Flag Football by player position.

	Quarterback (n = 12)	Wide Receiver (n = 38)	Centre (n = 13)	Defensive Back (n = 32)	Safety (n = 9)	Rusher (n = 5)
	n (%)	n (%)	n (%)	n (%)	n (%)	n (%)
Total injuries	9 (60)	28 (56)	10 (62.5)	27 (64.3)	8 (72.7)	5 (100)
ACL injuries	3 (20)	17 (34)	6 (37.5)	15 (35.7)	6 (54.5)	1 (20)
Shoulder injuries	3 (20)	14 (28)	2 (12.5)	11 (26.2)	1 (9.1)	3 (60)
Hand injuries	5 (33.3)	21 (42)	7 (43.8)	22 (52.4)	7 (63.6)	4 (80)

ACL = Anterior Cruciate Ligament.

**Table 3:** Main variables related to the incidence of injuries in Flag Football players.

	Odds Ratio	*p*	95% CI	
Preventive actions (No)	0.122	0.001	0.037	0.405
Relaxed arm girth (cm)	2.932	0.008	1.321	6.510
Flexed arm girth (cm)	0.335	0.009	0.149	0.757
Defensive back (Quarterback)	8.203	0.022	1.354	49.693
Tibiale mediale-sphyrion [tibiale length] (cm)	1.407	0.034	1.027	1.928
Muscle mass (%)	1.223	0.010	1.049	1.425

Injuries as dependent variable; significant differences *p* ≤ 0.05.

**Table 4:** Anthropometric variables associated with ACL injuries in Flag Football players.

	Odds Ratio	*p*	95% CI		
Relative leg length (cm)	0.447	0.012	0.238	0.836	0.447
Calf skinfold (<7 mm)	0.133	0.002	0.036	0.489	0.133
Hip girth (<103 cm)	5.689	0.010	1.518	21.325	5.689

Anterior Cruciate Ligament (ACL) injuries as dependent variable; significant differences *p* ≤ 0.05.

## Data Availability

The data presented in this study are available on https://doi.org/10.5281/zenodo.18224610, accessed on 21 March 2026.

## Supplementary Materials

The following supporting information can be downloaded at: https://www.mdpi.com/article/10.3390/sports14040140/s1, Injury questionnaire, Table S1: Descriptive characteristics of Flag Football elite players according to sex, Table S2: Univariate analysis of anthropometric variables, body composition and performance indexes in relation to injuries, Table S3. Anthropometric variables and body composition differences by sex.

## Abbreviations

The following abbreviations are used in this manuscript:

FF	Flag Football
TF	Tackle Football
ISAK	International Society of Advances for Kinanthropometry
ACL	Anterior Cruciate Ligament
AUC	Area Under the Curve
DEXA	Dual Energy X-ray Absorptiometry
BMI	Body Mass Index
COM	Centre of Mass
