# Supplementary material for: The Role of Anthropometry in Decision-Making for Injury Prevention Among Elite Flag Football Players
Source: Sports (Basel). 2026 Apr 1;14(4):140. doi: 10.3390/sports14040140 (PMC13119720; doi:10.3390/sports14040140)
Supplement: Supplementary file 1 [file sports-14-00140-s001.zip › Body composition and descriptive data.pdf]

Table S1. Descriptive characteristics of Flag Football elite players according to sex

		Total (N=139)		Males (n=91)		Females (n=48)	
		Frecuency	%	Frecuency	%	Frecuency	%
Ethnicity	Caucasian	120	86.3	79	86.8	41	85.4
	Mulato	3	2.2	1	1.1	2	4.2
	African	6	4.3	2	2.2	4	8.4
	Middle East	10	7.2	9	9.9	1	2.1
Nation	Great Britain	17	12.2	4	4.4	13	27.1
	Germany	11	7.9	11	12.1	0	0
	Ireland	24	17.3	12	13.2	12	25
	Spain	24	17.3	12	13.2	12	25
	France	23	16.5	12	13.2	11	22.9
	Georgia	6	4.3	6	6.6	0	0
	Israel	10	7.2	10	11	0	0
	Sweden	12	8.6	12	13.2	0	0
	Poland	12	8.6	12	13.2	0	0
Experience	< 3 years	29	26.9	18	24.3	11	32.4
	3 to 5 years	27	25	21	28.4	6	17.6
	5 to 7 years	16	14.8	9	12.2	7	20.6
	> 7 years	36	33.3	26	35.1	10	29.4
Player position	Quarterback	15	10.8	11	12.1	4	8.3
	Wide receiver	44	31.7	28	30.8	16	33.3
	Center	15	10.8	10	11	5	10.4
	Defensive back	33	23.7	21	23.1	12	25
	Safety	20	14.4	11	12.1	9	18.8
	Rusher	12	8.6	10	11	2	4.2

Table S2. Univariate analysis of anthropometric variables, body composition and performance indexes in relation to injuries

	Odds Ratio	*p*	95% IC	
Body Mass (kg)	1.035	0.081*	0.996	1.077
Stretch Stature (cm)	1.033	0.196*	0.983	1.085
Sitting Height (cm)	1.055	0.290	0.955	1.165
Arm Span (cm)	1.023	0.298	0.980	1.068
Triceps SF (mm)	0.961	0.294	0.893	1.034
Subscapular SF (mm)	1.068	0.279	0.947	1.205
Biceps SF (mm)	0.952	0.467	0.836	1.085
Iliac Crest SF (mm)	1.013	0.689	0.947	1.084
Supraspinale SF (mm)	1.039	0.431	0.943	1.146
Abdominal SF (mm)	1.036	0.308	0.967	1.109
Thigh SF (mm)	0.960	0.139*	0.911	1.013
Calf SF (mm)	0.940	0.101*	0.874	1.011
Head Girth (cm)	1.073	0.581	0.834	1.380
Neck Girth (cm)	1.110	0.127*	0.970	1.270
Arm Relaxed Girth (cm)	1.136	0.095*	0.977	1.320
Arm Flexed and Tensed Girth (cm)	1.103	0.146*	0.966	1.260
Forearm Girth (cm)	1.161	0.131*	0.956	1.410
Wrist Girth (cm)	1.093	0.625	0.763	1.565
Chest Girth (cm)	1.068	0.040*	1.002	1.138
Waist Girth (cm)	1.072	0.034*	1.005	1.143
Hips Girth (cm)	1.046	0.250	0.968	1.129
Thigh 1cm Gluteal Girth (cm)	1.063	0.276	0.951	1.275
Thigh Middle Girth (cm)	1.128	0.051*	0.999	1.275
Calf Girth (cm)	1.186	0.080*	0.979	1.436
Ankle Girth (cm)	1.377	0.056*	0.991	1.914
Acromiale-Radiale Length (cm)	1.063	0.569	0.859	1.316
Radiale-Stylion Length (cm)	1.178	0.197*	0.918	1.511
Midstylion-Dactylion Length (cm)	1.072	0.621	0.813	1.413
Iliospinale Height (cm)	1.070	0.084*	0.990	1.156
Trochanterion Height (cm)	1.062	0.183*	0.976	1.132
Trochanterion-Tibiale Laterale Length (cm)	1.057	0.359	0.938	1.190
Tibiale Laterale Height (cm)	1.047	0.459	0.925	1.186
Foot Length (cm)	1.319	0.049*	1.001	1.737
Tibiale Mediale-Sphyrion Tibiale Length (cm)	1.162	0.096*	0.973	1.387
Biacromial Breadth (cm)	1.097	0.243	0.939	1.281
Biiliocristal Breadth (cm)	1.053	0.604	0.865	1.282

Transverse Chest Breadth (cm)	1.176	0.108*	0.964	1.435
A-P Chest Depth (cm)	1.076	0.484	0.876	1.321
Humerus Breadth (cm)	1.537	0.282	0.702	3.365
Bi-Styloid Breadth (cm)	1.468	0.411	0.587	3.671
Femur Breadth (cm)	1.665	0.163*	0.813	3.408
Bimalleolar Breadth (cm)	1.850	0.114*	0.862	3.987
Muscle Mass (kg)	1.097	0.042*	1.003	1.200
Adipose Mass (kg)	0.936	0.106*	0.865	1.013
Bone Mass (kg)	1.126	0.413	0.847	1.497
Skin Mass (kg)	1.274	0.546	0.579	2.801
Residual Mass (kg)	1.265	0.070*	0.980	1.632
Adipose to muscular index (gr)	0.998	0.084*	0.995	1.000
Muscle to bone index (kg)	3.006	0.015*	1.243	7.270
Braquial index (cm)	1.101	0.176*	0.957	1.266
Crural index (cm)	1.015	0.631	0.953	1.082
Relative arm span (cm)	0.956	0.700	0.765	1.196
Acromio iliac index (cm)	0.973	0.559	0.888	1.066
Formic index (cm)	0.852	0.408	0.583	1.244

Injuries as dependent variable; * = potential association $p \leq 0.200$

Table S3. Anthropometric variables and body composition differences by sex

	Total (N=139)		Males (n=91)		Females (n=48)		
	Mean	SD	Mean	SD	Mean	SD	*p*
Body Mass (kg)	77.3	12.9	83.1	10.9	66.3	8.6	<0.001**
Stretch Stature (cm)	176.1	9.9	181.5	7.1	165.8	5.2	<0.001**
Sitting Height (cm)	92.8	4.8	95.3	3.6	88.2	3.1	<0.001**
Arm Span (cm)	178.5	11.2	184.8	7.7	166.7	6.0	<0.001**
Triceps SF (mm)	11.8	5.8	9.5	4.6	16.3	5.2	<0.001**
Subscapular SF (mm)	10.8	4.3	10.2	3.5	12.0	5.2	0.019*
Biceps SF (mm)	4.9	3.2	3.8	1.6	7.0	4.2	<0.001**
Iliac Crest SF (mm)	14.9	6.7	14.5	6.1	15.7	7.8	0.322
Supraspinale SF (mm)	9.4	5.2	8.7	4.1	10.8	6.7	0.025*
Abdominal SF (mm)	16.3	7.1	16.3	6.7	16.4	8.0	0.885
Thigh SF (mm)	16.1	8.3	12.6	6.1	22.7	7.8	<0.001**
Calf SF (mm)	10.2	5.8	7.9	4.0	14.6	6.2	<0.001**
Head Girth (cm)	56.3	1.8	56.9	1.6	55.2	1.7	<0.001**
Neck Girth (cm)	36.4	3.4	38.4	1.8	32.6	2.2	<0.001**
Arm Relaxed Girth (cm)	32.0	3.1	33.4	2.3	29.3	2.6	<0.001**
Arm Flexed and Tensed Girth (cm)	33.4	3.5	35.3	2.3	29.8	2.3	<0.001**
Forearm Girth (cm)	27.4	2.4	28.8	1.3	24.9	1.6	<0.001**
Wrist Girth (cm)	16.4	1.3	17.2	0.9	15.1	0.7	<0.001**
Chest Girth (cm)	98.1	7.8	102.0	5.8	90.7	5.1	<0.001**
Waist Girth (cm)	80.5	7.7	83.8	6.0	74.2	6.3	<0.001**
Hips Girth (cm)	100.8	6.3	101.3	6.2	99.9	6.4	0.198
Thigh 1cm Gluteal Girth (cm)	61.2	4.2	61.5	4.0	60.7	4.4	0.284
Thigh Middle Girth (cm)	55.6	3.9	56.3	3.5	54.1	4.1	0.001*
Calf Girth (cm)	38.0	2.4	38.6	2.4	37.0	2.0	<0.001**
Ankle Girth (cm)	22.8	1.5	23.2	1.3	21.8	1.5	<0.001**
Acromiale-Radiale Length (cm)	33.9	2.2	34.9	1.7	31.9	1.7	<0.001**
Radiale-Stylion Length (cm)	26.0	1.8	26.9	1.4	24.3	1.3	<0.001**
Midstylion-Dactylion Length (cm)	19.5	1.6	20.1	1.2	18.2	1.4	<0.001**
Iliospinale Height (cm)	98.5	6.5	101.8	5.1	92.2	3.8	<0.001**
Trochanterion Height (cm)	92.1	6.5	95.1	5.6	86.4	3.9	<0.001**
Trochanterion-Tibiale Laterale Length (cm)	45.2	5.4	45.9	3.7	44.0	7.5	0.042*
Tibiale Laterale Height (cm)	47.3	3.8	49.2	2.9	43.6	2.3	<0.001**
Foot Length (cm)	26.1	1.8	27.0	1.3	24.3	1.1	<0.001**
Tibiale Mediale-Sphyrion Tibiale Length (cm)	39.4	2.8	40.7	2.4	37.0	1.8	<0.001**
Biacromial Breadth (cm)	40.5	3.0	42.1	2.3	37.6	1.6	<0.001**

Biiliocristal Breadth (cm)	28.9	2.4	29.4	2.5	28.1	2.0	0.002*
Transverse Chest Breadth (cm)	29.2	2.6	30.3	2.5	27.1	1.4	<0.001**
A-P Chest Depth (cm)	19.0	2.3	19.9	1.9	17.3	1.9	<0.001**
Humerus Breadth (cm)	7.0	0.6	7.3	0.4	6.4	0.3	<0.001**
Bi-Styloid Breadth (cm)	5.7	0.5	5.9	0.4	5.2	0.3	<0.001**
Femur Breadth (cm)	9.8	0.7	10.1	0.6	9.3	0.5	<0.001**
Bimalleolar Breadth (cm)	7.3	0.6	7.6	0.5	6.6	0.3	<0.001**
Muscle Mass (kg)	36.0	7.4	40.4	4.6	27.8	4.0	<0.001**
Adipose Mass (kg)	19.8	5.1	19.5	5.2	20.5	4.8	0.275
Bone Mass (kg)	8.8	1.7	9.5	1.5	7.5	1.0	<0.001**
Skin Mass (kg)	3.9	0.5	4.1	0.5	3.7	0.3	<0.001**
Residual Mass (kg)	8.7	1.9	9.6	1.5	6.8	1.1	<0.001**
% of Muscle Mass	46.5	4.9	48.8	3.7	42.1	3.9	<0.001**
% of Adipose Mass	25.8	5.5	23.2	3.8	30.7	4.7	<0.001**
% of Bone Mass	11.4	1.0	11.5	0.9	11.3	1.2	0.553
% of Skin Mass	5.2	0.7	5.0	0.6	5.6	0.5	<0.001**
% of Residual Mass	11.1	1.0	11.6	0.8	10.3	0.7	<0.001**
Summation of 8 Skinfolds (mm)	94.6	37.5	83.5	30.5	115.5	40.9	<0.001**
Summation of 6 Skinfolds (mm)	74.7	30.4	65.2	24.8	92.8	32.0	<0.001**

* = significative differences in t-student test $p \leq 0.05$, **= significant differences in t-student test $p \leq 0.01$.
