# Supplementary material for: The Role of Anthropometry in Decision-Making for Injury Prevention Among Elite Flag Football Players
Source: Sports (Basel). 2026 Apr 1;14(4):140. doi: 10.3390/sports14040140 (PMC13119720; doi:10.3390/sports14040140)
Supplement: Supplementary file 1 [file sports-14-00140-s001.zip › Injury questionnaire.pdf]

Flag Football Players in the IFAF 2023 European Championships Research Project: Injury questionnaire

We are very thankful with you for taking a few minutes to fill this information that is very important for the development of this research study. All the answers will be processed under confidential statements.
Please take your time to read and understand all the questions, if you have any doubt please let us know.

All data will be collected anonymously using unique participant codes – participants will receive a number at the start of the study. All data collected for that participant will be filed under this number and data collection will be anonymous. There will be one file with the number and name of the participants and this will be locked and stored with a password with access only to the research team members.

1. What is your national team?

- Sweeden
- Israel
- Switzerland
- Denmark
- Germany
- Serbia
- Finlandia
- Spain
- Croatia
- Italy
- Belgium
- France
- Luxemburg
- Czech
- Norway
- Russia
- Belarus
- Slovakia
- Ireland
- Poland
- Great Britain
- Nigeria
- Cameroon
- Georgía

2. Project Personal Identification Number

____

3. Sex

- Male
- Female

4. What is your main position in the game?

- Quarterback
- Wide receiver
- Center
- Defensive Back
- Safety
- Rusher

5. How many years have you practised Flag Football?
Take in to account the years of formal training

- Less than 3 years
- Between 3 to 5 years
- Between 5.1 to 7 years
- More than 7 years

6. What is the frequency of your isolated physical training sessions per week?

- I am not participating in any other physical training sessions besides Flag Football
- Less than 3 sessions by week
- 3 to 5 sessions by week
- More than 5 sessions by week
- Also I use to practice other formal sport

7. On average, how long do your training sessions typically last?

- Less than 60 minutes per session
- Between 60 to 120 minutes per session
- More than 120 minutes per session

8. How often do you take preventive measures to avoid injuries? Some examples of these measures could include ice baths, cryotherapy, or other forms of physical therapy.

- I don't use to take any actions to prevent injuries
- I use to take this kind of actions at least once a month
- I use to take this kind of actions at least twice in a month
- I use to take this kind of actions at least once a week
- I use to take this kind of actions at least more than once in a week
- Prefer not to say

9. In the last five years, have you experienced any knee injuries?

- None
- Meniscal injuries
- Collateral sprain
- Bursitis
- ACL (Anterior cruciate ligament) sprain or rupture
- Tick this box if any of these injuries required surgery
- Other _____________

10. In the last 5 years, have you had any shoulder injury?

- None
- Biceps tendinopathy
- Rotator cuff tendinopathy or rotator cuff muscle strain
- Bursitis
- Shoulder dislocation
- Clavicle sprain or fracture
- Tick this box if any of these injuries required surgery
- Other ______________

11. In the last 5 years, have you had any hand injuries?

- None
- Finger sprain
- Finger dislocation
- Carpal bones dislocation
- Fractures
- Mark here if any of these injuries required surgery
- Other _____________

12. Finally, have you had any other injuries associated with flag football in the last 10 years? If YES, please tell us the kind of injury

____
